# Serelaxin improves cardiac and renal function in DOCA-salt hypertensive rats

**DOI:** 10.1038/s41598-017-09470-0

**Published:** 2017-08-29

**Authors:** Dong Wang, Yuhuan Luo, Komuraiah Myakala, David J. Orlicky, Evgenia Dobrinskikh, Xiaoxin Wang, Moshe Levi

**Affiliations:** 1 Renal Diseases and Hypertension, School of Medicine, University of Colorado ANSCHUTZ MEDICAL CAMPUS, Aurora Colorado, 80045 USA; 2 Department of Pathology, School of Medicine, University of Colorado ANSCHUTZ MEDICAL CAMPUS, Aurora Colorado, 80045 USA

## Abstract

Serelaxin, a recombinant form of the naturally occurring peptide hormone relaxin-2, is a pleiotropic vasodilating hormone that has been studied in patients with acute heart failure. In this study, the effects of serelaxin on cardiac and renal function, fibrosis, inflammation and lipid accumulation were studied in DOCA-salt treated rats. Uninephrectomized rats were assigned to two groups: controls provided with normal drinking water and DOCA provided with DOCA pellets and sodium chloride drinking water. After 4 weeks, the DOCA-salt rats were randomly selected and implanted with osmotic minipumps delivering vehicle or serelaxin for another 4 weeks. Treatment with serelaxin prevented cardiac and renal dysfunction in DOCA-salt rats. Serelaxin prevented cardiac and renal fibrosis, as determined by Picrosirius Red staining and Second Harmonic Generation (SHG) Microscopy. Treatment of DOCA-salt rats with serelaxin decreased renal inflammation, including the expression of TGF-β, NFκB, MCP-1, IL-**1**, IL-**6**, ICAM-1, VCAM-1 and CD68 macrophages. Serelaxin also decreased lipid accumulation in kidney in part by decreasing SREBP-1c, SREBP-2, ChREBP, FATP1, HMGCoAR, and LDL receptor, and increasing Acox1 and ABCA1. In summary, serelaxin reversed DOCA-salt induced cardiac and renal dysfunction.

## Introduction

Chronic kidney disease (CKD) affects more than 10% of the population worldwide and its prevalence is increasing^1^. It consists of a diverse range of etiologies, including immunological, mechanical, metabolic and toxic insults, among others. These variously affect the three functional compartments of the kidney, the vasculature, glomerulus and the tubulointerstitium. The patients with CKD show a progressive decline in renal function with time. The process is irreversible, inevitably leading to end-stage renal failure^2^.

Relaxin is a 6 kDa peptide hormone which mediates many adaptive hemodynamic changes that occur during pregnancy, such as increased cardiac output, increased renal blood flow, and increased arterial compliance^3^. In the female, it is produced by the corpus luteum of the ovary and breast during pregnancy, also by the placenta, chorion, and decidua^4^. In the male, it is produced in the prostate and is present in human semen^5^. The relaxin-like peptide family consists of 7 peptides of high structural but low sequence similarity; relaxin-1 (RLN1), 2 (RLN2) and 3 (RLN3), and the insulin-like (INSL) peptides, INSL3, INSL4, INSL5 and INSL6^6^.

Serelaxin, a recombinant form of the human peptide relaxin-2, is a pleiotropic vasodilating hormone that has been studied in patients with acute heart failure^7^. It mediates vasodilation by increasing the production of nitric oxide (NO), a potent vasodilator. Activation of G-protein-coupled receptor RXFP1 activates several enzymes in a phosphorylation cascade (cAMP, ERK-1/2) that eventually results in the activation of NO synthase in endothelial cells and the subsequent production of NO. Relaxin inhibits renal myofibroblast differentiation through RXFP1, the nitric oxide pathway, and the inhibition of α-SMA and phosphorylation of Smad2^8^. Beside these effects, relaxin can also bind to endothelial B receptor on endothelial cells to induce vasodilation^9^.

In hypertensive rats, relaxin treatment ameliorates ventricular hypertrophy and fibrosis by reducing cardiac collagen content, leading to improved cardiac function^10–12^. Serelaxin attenuates myocardial ischemia/reperfusion (I/R) injury and the subsequent caspase-1 activation via an eNOS-dependent mechanism^13^. Relaxin also attenuates myocardial injury and preserves cardiac function by alleviating cardiac fibrosis and ventricular dysfunction in an isoproterenol-induced myocardial ischemic rat model^14^. Although a majority of the studies involving serelaxin have focused on the heart, serelaxin increases creatinine clearance, renal blood flow, urine flow and sodium excretion in the kidney^15–17^. Serelaxin is also a potential treatment for renal dysfunction in cirrhosis^18^.

Based on these findings, the current study was designed to determine the protective role of serelaxin on cardiac and renal function in deoxycorticosterone acetate (DOCA)-salt hypertension rats. Our studies indicate that serelaxin treatment reversed cardiac dysfunction, left ventricular hypertrophy, and cardiac fibrosis. Furthermore, serelaxin also decreased renal dysfunction, fibrosis, inflammation and lipid accumulation. Thus, these results firmly establish an important role of serelaxin in preventing cardiac and kidney dysfunction in the DOCA-salt hypertensive model of cardiorenal disease.

## Results

### Serelaxin effects on metabolic parameters

There were no effects of the serelaxin administration on food intake and body weight throughout the treatment period, and relative liver weight and tibia length were similarly unchanged. Although kidney and heart weights were slightly but significantly increased, 4 weeks of treatment with serelaxin did not reduce the increases in kidney and heart weights. The systolic blood pressure was increased in DOCA-salt rats compared with control rats, whereas serelaxin normalized the increased systolic blood pressure in DOCA-salt rats at 4 weeks after DOCA implantation^19, 20^ (Table 1).

**Table 1:** Physiological parameters.

	Control	DOCA-salt rats	Serelaxin treated DOCA-salt rats
Body weight	481.50 ± 11.87	473.00 ± 28.44	479.83 ± 15.41
Kidney weight	2.04 ± 0.08	2.87 ± 0.27*	2.54 ± 0.15
Liver weight	14.05 ± 0.76	14.54 ± 0.78	13.91 ± 0.82
Heart weight	1.17 ± 0.06	1.55 ± 0.07*	1.53 ± 0.09
Tibia length	4.32 ± 0.03	4.30 ± 0.04	4.29 ± 0.04
Blood pressure	108.19	154.86*	110.08**

All values shown represent the mean ± SEM. N = 6 rats per group. Significance: *P < 0.05 vs Control rats; **P < 0.05 vs DOCA-salt rats.

### Serelaxin improves cardiac function

DOCA-salt rats exhibited marked cardiovascular remodeling, including increased a) left ventricular internal diameter end diastole, b) left ventricular internal diameter end systole, c) increased left ventricular posterior wall end diastole, d) left ventricular posterior wall end systole, e) LV systolic volume, f) LV diastolic volume, and g) increased Interventricular septal (Table 2). Treatment with serelaxin in the DOCA-salt rats prevented most but not all the abnormalities.

**Table 2:** Cardiac Echo Measurements.

	Control	DOCA-salt rats	Serelaxin treated DOCA-salt rats
IVSd (mm)	1.28 ± 0.03	1.53 ± 0.06*	1.33 ± 0.05**
IVSs (mm)	2.00 ± 0.05	2.22 ± 0.07*	2.12 ± 0.09
LVIDd (mm)	7.74 ± 0.17	8.58 ± 0.15*	8.22 ± 0.17
LVIDs (mm)	4.61 ± 0.18	5.42 ± 0.28*	4.79 ± 0.24
IVPWd (mm)	1.28 ± 0.03	1.52 ± 0.06*	1.32 ± 0.05**
LVPWs (mm)	1.99 ± 0.04	2.23 ± 0.07*	2.11 ± 0.09
Heart Rate (BPM)	373.15 ± 6.31	354.36 ± 7.25	405.30 ± 12.04**
LV Vold (ul)	321.65 ± 16.35	403.55 ± 15.46*	367.36 ± 16.90
LV Vols (ul)	99.07 ± 9.60	145.22 ± 16.56*	108.83 ± 12.97
Ejection fraction (%)	69.52 ± 1.58	64.00 ± 3.83	70.79 ± 2.32

All values shown represent the mean ± SEM. N = 6 rats per group. IVSd (Interventricular septal end diastole), IVSs (Interventricular septal end systole), LVIDd (Left ventricular internal diameter end diastole), LVIDs (Left ventricular internal diameter end systole), LVPWd (Left ventricular posterior wall end diastole), LVPWs (Left ventricular posterior wall end systole), LV Vold, (LV diastolic volume), LV Vols (LV systolic volume). Significance: *P < 0.05 vs Control rats; **P < 0.05 vs DOCA-salt rats.

**Figure 1:**
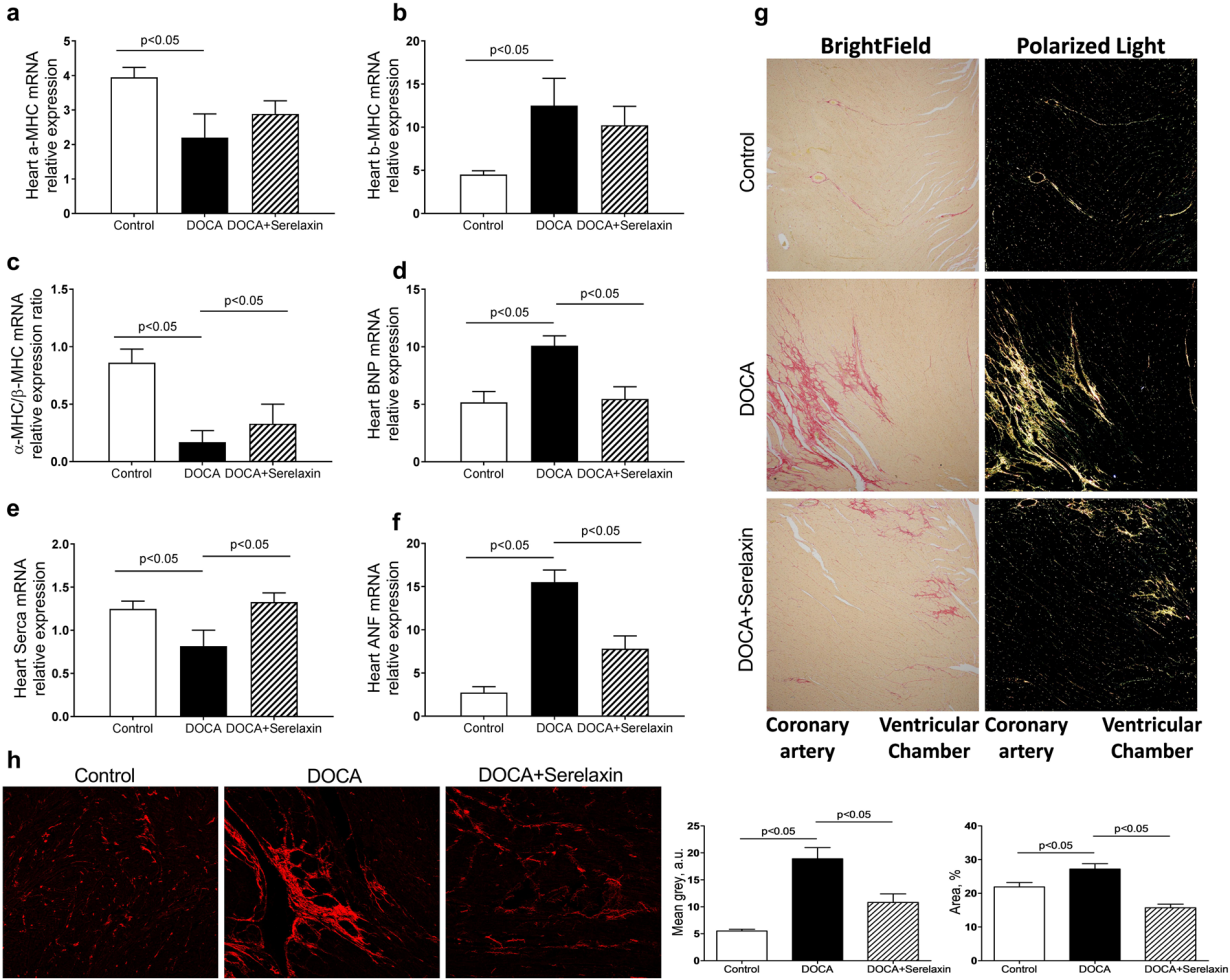
Serelaxin improved heart function of DOCA-salt rats. (**a**–**f**) Markers of cardiac hypertrophy and dysfunction as determined by quantitative real-time PCR (qRT-PCR) analysis of α-MHC, β-MHC, BNP, ANF, SERCA in the heart of DOCA-salt rats. Treatment with serelaxin increased α-MHC/β-MHC, decreased BNP and ANF, and increased SERCA. (**g**) Cardiac fibrosis determined by Picro Sirius Red staining for fibrillary collagens. DOCA induced cardiac fibrosis and treatment with serelaxin prevented the cardiac fibrosis. **(h**) Cardiac fibrosis as determined by Second Harmonic Generation (SHG) Microscopy showed DOCA induced a marked increase in cardiac fibrosis and that treatment with serelaxin markedly decreased the cardiac fibrosis. Results are expressed as means ± SEM (n = 6 rats). Statistical analysis was performed with one-way ANOVA.

Alpha-myosin heavy chain (α-MHC), beta-myosin heavy chain (β-MHC), brain natriuretic peptide (BNP), atrial natriuretic factor (ANF) and sarco/endoplasmic reticulum Ca-ATPase (SERCA) are commonly used as biomarkers of cardiac hypertrophy^21–24^. In DOCA-salt rats, there was a significant decrease in α-MHC mRNA compared with UNX rats, whereas in serelaxin-treated rats, there was a trend toward an increase that did not achieve significance (Fig. 1a). β-MHC expression was significantly increased in DOCA-salt rats but reduced in the presence of serelaxin (Fig. 1b). An analysis of α-MHC/β-MHC expression ratio demonstrated a five-fold decrease in DOCA-salt rats compared with control rats, whereas a two-fold increase following treatment with serelaxin (Fig. 1c). The expression of BNP and ANF mRNA were increased in DOCA-salt rats, which were attenuated by serelaxin treatment, while the expression of SERCA was decreased in DOCA-salt rats, and the decrease was reversed by serelaxin treatment (Fig. 1d,e,f).

### Serelaxin decreases cardiac fibrosis

There was a dramatic increase in interstitial and perivascular myocardial collagen in the subendocardial and midmyocardial regions of the left ventricle of DOCA-salt rats as determined by picrosirius red staining. Treatment with serelaxin showed a decreased higher order assembly of collagens as determined by decreased fibrillary collagen presence evaluated by picrosirius red staining and orthogonal light interrogation, indicating that serelaxin attenuated and possibly reversed fibrillary collagen deposition in the left ventricle of DOCA-salt rats (Fig. 1g).

Highly ordered fibrillary collagens (types I and III) produce SHG signals, which can be visualized in tissue without the need for exogenous labeling. To further determine fibrosis of the heart, label-free and stain-free imaging with Two Photon Excitation (TPE)-Second Harmonic Generation (SHG) Microscopy was then used to visualize the fibrillary structure in paraffin-embedded and unstained heart sections^25, 26^. SHG revealed a decrease in fibrillary collagen accumulation in serelaxin treated DOCA-salt rats (Fig. 1h). In summary, these results demonstrated that there was fibrotic changes and functional impairment in DOCA-salt hypertensive rats, and the treatment with serelaxin for 4 weeks was able to reverse many of these pathologic changes.

### Serelaxin decreases renal dysfunction

**Figure 2:**
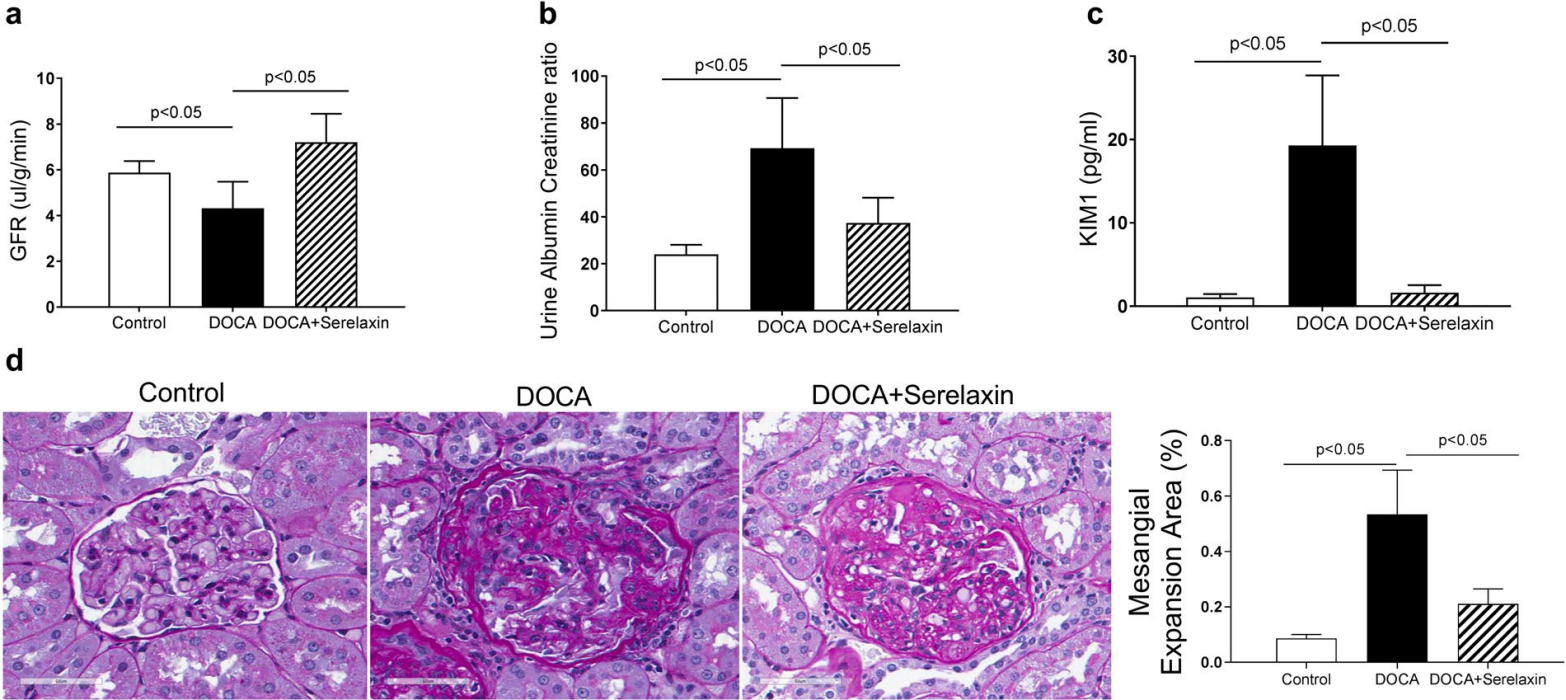
Serelaxin improved renal function of DOCA-salt rats. (**a**) DOCA induced a decrease in glomerular filtration rate and treatment with serelaxin reversed the decrease in glomerular filtration rate. (**b**) DOCA markedly increased urine albumin/creatinine excretion rate, and treatment with serelaxin significantly decreased urine albumin/creatinine excretion rate. **(c)** DOCA induced a marked increase in urinary KIM1 excretion and treatment with serelaxin significantly decreased and normalized KIM1 excretion. (**d**) DOCA increased glomerular extracellular matrix accumulation and mesangial expansion, as determined by Periodic acid–Schiff (PAS) staining, and treatment with serelaxin markedly decreased the matrix accumulation and mesangial expansion. Results are expressed as means ± SEM (n = 6 rats). Statistical analysis was performed with one-way ANOVA.

DOCA induced a decrease in glomerular filtration rate (GFR) and treatment with serelaxin reversed the decrease (Fig. 2a). Similarly, the urinary albumin/creatinine ratio (Fig. 2b) and the excretion of urinary KIM-1 (Fig. 2c), which is a marker of tubular damage^27^, were significantly higher in DOCA-salt rats compared with control rats. Serelaxin fully blocked all these effects (Fig. 2b,c). In addition, DOCA increased glomerular extracellular matrix accumulation and mesangial expansion, as determined by PAS staining, and treatment with serelaxin markedly attenuated those increases (Fig. 2d).

### Serelaxin decreases renal fibrosis

**Figure 3:**
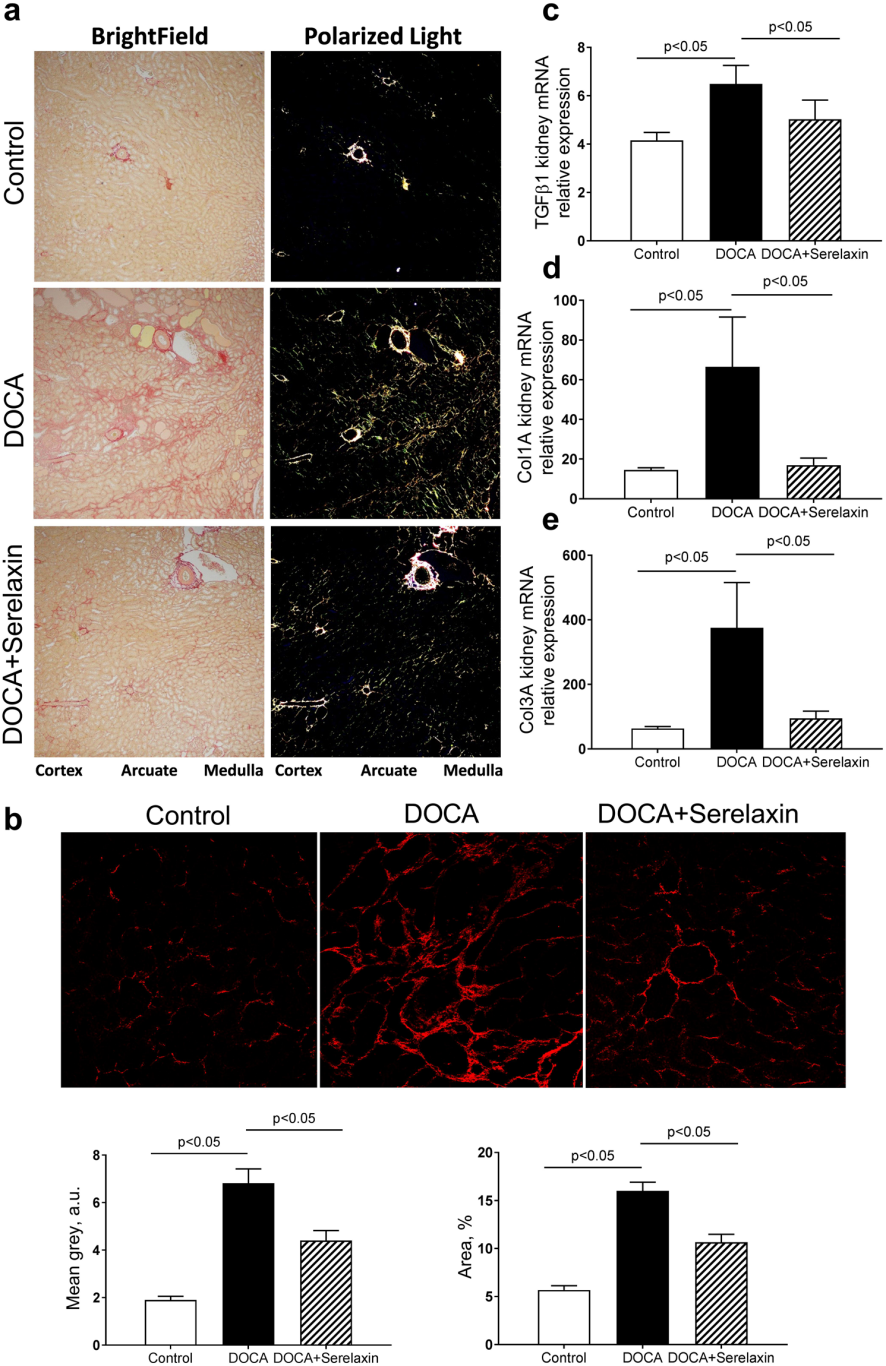
Serelaxin decreased kidney fibrosis of DOCA-salt rats. (**a**) DOCA increased periglomerular and tubulointerstitial fibrosis, as determined by Picrosirius Red staining, and treatment with serelaxin markedly decreased the periglomerular and tubulointerstitial fibrosis. (**b**) Second Harmonic Generation (SHG) Microscopy showed DOCA induced a marked increase in tubulointerstitial fibrosis and treatment with serelaxin markedly decreased the fibrosis. Quantitative real-time PCR (qRT-PCR) analysis showed treatment with serelaxin prevented the increases in the **(c)** profibrotic factor TGF-β, (**d**) collagen1A and (**e**) collagen 3 A gene expression in the kidney. Results are expressed as means ± SEM (n = 6 rats). Statistical analysis was performed with one-way ANOVA.

In the kidney, DOCA treatment increased the collagen around the arcuate, interlobular arteries, and around many of the tubules in the outer stripe of the outer medulla. Serelaxin treated rats appeared to have only a slightly decreased Picro-Sirius detectable collagen content when compared to the DOCA group, but closer inspection revealed that the medullary peri-tubular collagen did not have the same pattern as observed in the DOCA only treated group. Serelaxin appeared to disrupt the collagen and started to break it into smaller picrosirius red positive pieces (Fig. 3a).

While, DOCA induced a marked increase in tubulointerstitial fibrosis (red signal) and treatment with serelaxin markedly decreased the fibrosis, as determined by TPE-SHG microscopy (Fig. 3b).

These pathologic improvements were accompanied by serelaxin induced decreases in the expression of the profibrotic growth factor TGF-β (Fig. 3c) and the profibrotic collagens Col1A and Col3A mRNA (Fig. 3d and e).

### Serelaxin decreases renal inflammation

**Figure 4:**
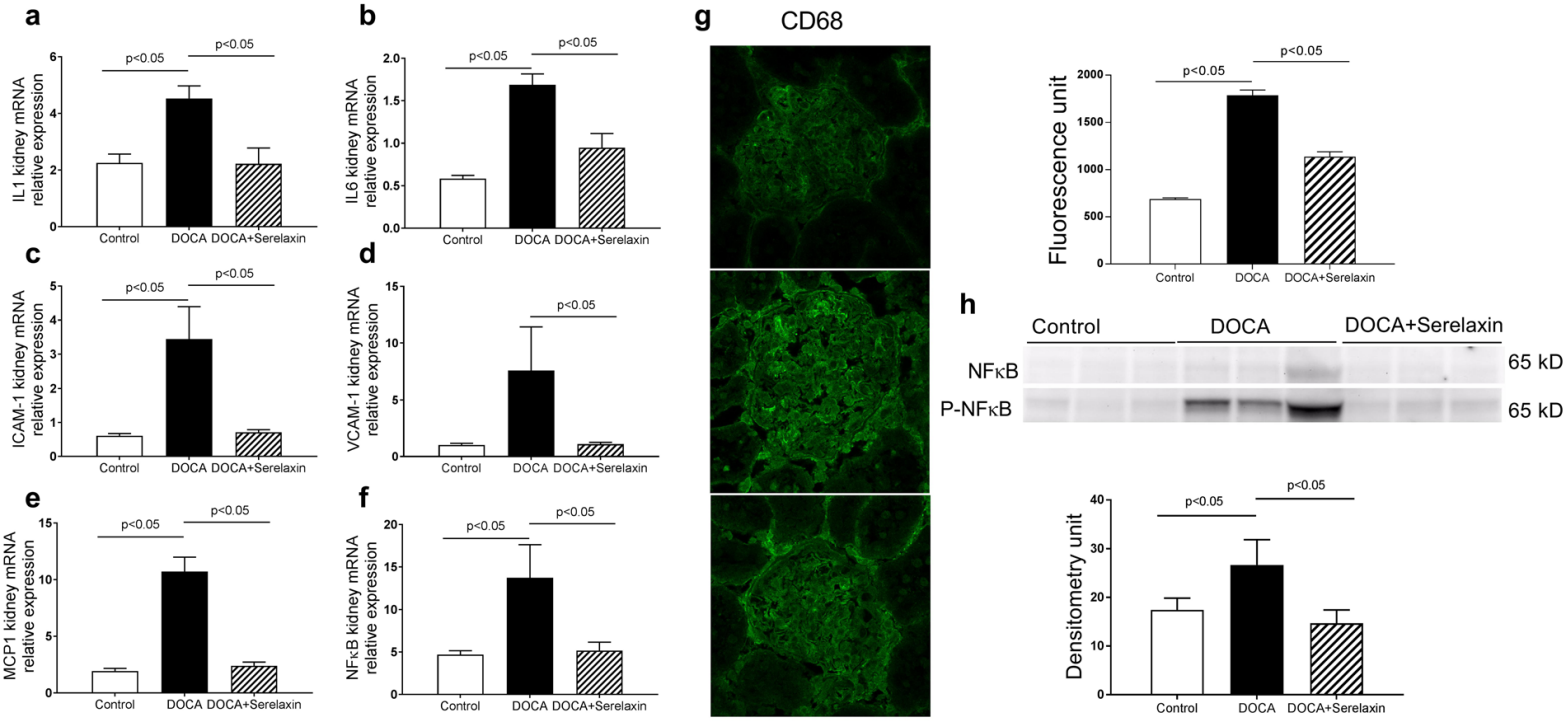
Serelaxin decreased kidney inflammation. Quantitative real-time PCR (qRT-PCR) analysis showed that treatment with serelaxin prevented the increases in the inflammatory markers (**a**) IL-1-β, (**b**) IL-6, (**c**) ICAM-1, (**d**) VCAM-1, (**e**) MCP-1, and (**f**) NFκB mRNA. Serelaxin also prevented the increases in (**g**) CD68 immunostaining and (**h**) phospho-NFκB protein abundance in the kidney. Equal protein loading was verified by using an anti-β-actin antibody. The detection was executed by an ECL western blotting analysis system. Whole bolts are reported in Supplemental Fig. 1. Results are expressed as means ± SEM (n = 6 rats for qRT-PCR, n = 3 for western blot). Statistical analysis was performed with one-way ANOVA.

There were significant increases in the mRNA abundance of interleukin-1β, interleukin-6, intercellular adhesion molecule-1 (ICAM-1), vascular cell adhesion molecule-1 (VCAM-1), monocyte chemotactic protein-1 (MCP-1), nuclear factor kappa-light-chain-enhancer of activated B cells (NFκB) (Fig. 4a–f) and CD68 positive macrophage immunostaining (Fig. 4g) in DOCA-salt rats compared to controls. The expression of these genes and immunoreactivity were markedly decreased in serelaxin treated rats. NFκB is a transcription factor that has crucial roles in inflammation, immunity, cell proliferation and apoptosis. Serelaxin modulated the NFκB signaling pathway by regulating phosphorylation of NFκB (Fig. 4h).

### Serelaxin decreases lipid accumulation in the kidney

**Figure 5:**
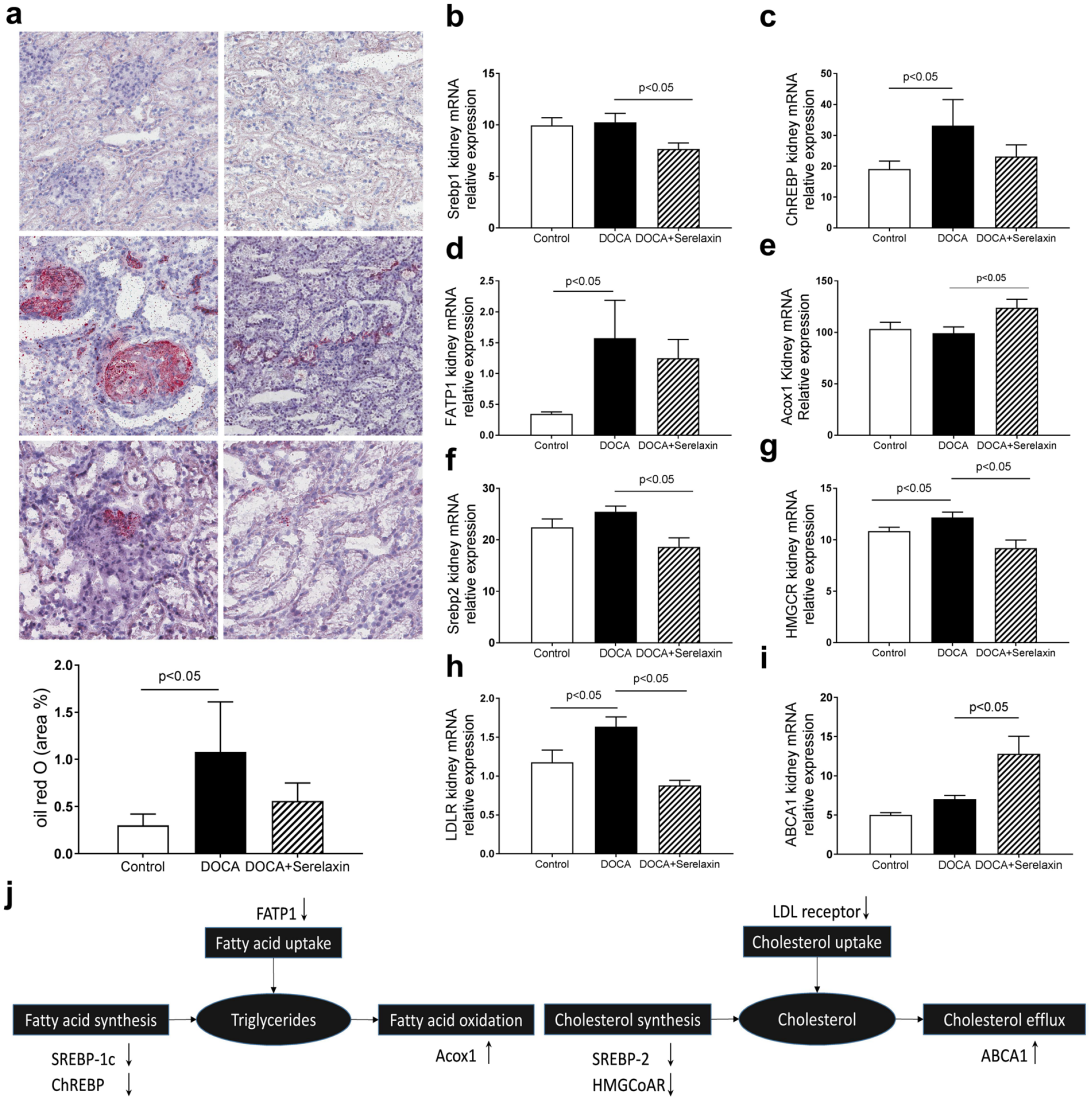
Serelaxin decreased kidney lipid accumulation. (**a**) Representative images of oil red O staining (20 X), which staining for neutral lipids, showed that increased neutral lipid accumulation in kidney of DOCA-salt rats was prevented by the treatment with serelaxin. The expression of fatty acid, triglyceride and cholesterol metabolism related genes were determined by quantitative real-time PCR (qRT-PCR) analysis, (**b**) SREBP-1c, (**c**) ChREBP, (**d**) FATP1, (**e**) Acox1, (**f**) SREBP-2, (**g**) HMGCR, (**h**) LDLR, (**i**) ABCA1. (**j**) Serelaxin regulated the genes involved in fatty acid, triglyceride, and cholesterol metabolism in DOCA-salt rats. Results are expressed as means ± SEM. Statistical analysis was performed with one-way ANOVA.

Unexpectedly DOCA-salt rats had increased renal neutral lipid accumulation in the glomeruli and tubulointerstitial cells as determined by increased oil red o (ORO) staining. There was a decrease trend of lipid accumulation in serelaxin treatment group (Fig. 5a). Increased triglyceride accumulation can occur because of a) increased fatty acid synthesis, b) increased fatty acid uptake, or c) decreased fatty acid oxidation. Increased cholesterol accumulation can occur because of i) increased cholesterol synthesis, ii) increased cholesterol uptake, or iii) decreased cholesterol efflux^28^. The expression of fatty acid, triglyceride and cholesterol metabolism related genes were measured in three groups. Treatment with serelaxin decreased the transcript levels of SREBP-1c, ChREBP, which are master regulators of fatty acid synthesis^29, 30^ and decreased the transcript levels of FATP1, which is mediator of fatty acid uptake, (Fig. 5b,c,d), and increased Acox1 that mediates fatty acid oxidation (Fig. 5e). Serelaxin also decreased the transcript levels of SREBP-2 and HMGCoAR, which are master regulators of cholesterol synthesis^31, 32^, and decreased LDL receptor (Fig. 5f,g,h), which is a mediator of cholesterol uptake and increased ABCA1 that mediates cholesterol efflux (Fig. 5i).

## Discussion

The heart and kidney are one of the prime organs of hypertensive damage. Uncontrolled hypertension accelerates the damage to these targets and results in eventual organ failure and cardiovascular death and disability^33^. In this study, Serelaxin significantly decreased the systolic blood pressure in DOCA-salt hypertensive rat. Thus, the blood pressure reduction may partially contribute to the protection of heart and kidney. However, in non-hypertension model, there are preclinical and clinical data demonstrated the impact of serelaxin treatment on heart and kidney function and protection. For example, in a pig models of ischemia-reperfusion (IR) injury, serelaxin treatment reduced cardiomyocyte damage and cardiac^34^. In rats subjected to IR injury, treatment of serelaxin can increase GFR, renal blood flow, protected against renal ischemia-reperfusion injury and glomerular dysfunction^35–38^. In healthy subjects, serelaxin can also increase renal blood flow compared with placebo^39^. Furthermore, in isoprenaline induced cardiac injury mice, serelaxin treatment reduced cardiac fibrosis to a greater than enalapril alone without change of blood pressure^10^. These findings suggest that the effects of serelaxin on heart and kidney are independent of blood pressure.

In contrast to other vasodilators which primarily act via direct venodilation^40^, the vasorelaxatory action of serelaxin is thought to mainly affect arteries^41^. There are studies suggests that serelaxin can increase arterial compliance and decrease systemic vascular resistance^16, 42^, which could mitigate hemodynamic abnormalities. Serelaxin acts via multiple molecular mechanisms to stimulate vasorelaxatory systems and relieve congestion. One possible mechanism of action of serelaxin is as a mediator of vasoactive system by increasing the production of nitric oxide (NO), a potent vasodilator^43^. By activating relaxin receptor RXFP1, which is a G protein coupled receptor, serelaxin promotes the synthesis of NO through several enzymes in phosphorylation cascade^44^. Besides this, serelaxin also causes vasodilation by an indirect mechanism, where it inhibits the potent vasoconstrictors angiotensin II and endothelin^45^.

This study demonstrates that serelaxin reversed the DOCA-salt induced renal dysfunction, including a decrease in GFR, an increase in urinary albumin, an increase in urinary KIM-1, and an increase in mesangial expansion. These data suggest serelaxin affects a several pathophysiological processes, which result in both haemodynamic abnormalities and end-organ damage. Furthermore, the serelaxin-induced ameliorations in renal function were accompanied by prevention or reversal of kidney fibrosis, as well as decreases in the profibrotic growth factor TGF-β and the expression of fibrillary collagens type 1 and type 3 collagen mRNAs. It is well known that TGF-β1 signal transduction mediates fibrosis progression by stimulating myofibroblast differentiation and collagen synthesis and deposition, while reducing collagen degradation^46^. The possible mechanism of serelaxin inhibits fibrosis is to interfere with TGF-β1 signaling via a mechanism of NO pathway (nNOS, NO, cGMP and Smad2)^8^. The inhibition of TGF-β1 reduces collagen deposition which results in inhibiting fibrosis of organ.

Inflammatory activation can also contribute to organ injury, vascular dysfunction and fluid overload^47, 48^. In current study, the DOCA-salt rat exhibited a marked increase in renal inflammation as determined by increased expression of proinflammatory cytokines, increased expression of the macrophage marker CD68 and increased abundance of the proinflammatory transcription factor NF-κB. Treatment with serelaxin prevented all the changes, thus suppressing the inflammatory state of the kidney. This anti-inflammatory action of serelaxin differentiate it from current other heart failure therapies, such as nitrates, which have not been shown to inhibit inflammation^49^.

Another finding from our study was the marked increase in renal lipid accumulation in the DOCA salt rat. There was a trend for serelaxin to decrease the renal lipid accumulation by change the expression of genes that mediate fatty acid synthesis (SREBP-1c and ChREBP), cholesterol synthesis (SREBP-2, HMGCoAR), fatty acid uptake (FATP1) and cholesterol uptake (LDLR), as well as fatty acid oxidation (Acox1) and cholesterol efflux (ABCA1)^28^.

Lastly, in a model of cardiorenal syndrome, rats with uninephrectomy treated with DOCA pellets and saline drinking water (DOCA-salt rats) for 8 weeks, including treatment (intervention) with serelaxin during weeks 5–8, we demonstrated the highly efficacious effects of serelaxin in reversing cardiac and renal structural, functional and metabolic alterations. We identified the metabolic actions of serelaxin in the heart and kidney which resulted in amelioration of ventricular hypertrophy and cardiac fibrosis, improvement of GFR, decrease of albuminuria and decreased in urinary excretion of kidney injury marker KIM-1, prevention of kidney inflammation and fibrosis, and decrease of lipid accumulation of kidney. This study suggests that serelaxin may provide organ protection via inhibition of inflammation and tissue fibrosis to improve the prognosis of patients with heart failure and kidney disease.

## Methods

### Animals

Eight weeks old uninephrectomized (UNX) Sprague Dawley rats were purchased from Charles River Laboratories. The rats were subsequently assigned to one of two groups: (i) UNX controls provided with normal drinking water (UNX) (n = 12); (ii) deoxycorticosterone acetate (DOCA) and sodium chloride (DOCA-salt) rats receiving 60-day time release DOCA pellets (50 mg; Innovative Research of America, Sarasota, FL) and 0.9% NaCl and 0.2% KCl in drinking water for 4 weeks (UNX + DOCA) (n = 24). Then DOCA-salt rats were randomly selected and implanted with osmotic minipumps delivering a) vehicle (N = 12) or b) 16 mg/kg/day Serelaxin (N = 12) for another 4 weeks. When the rats were 16 weeks old, blood pressure, glomerular filtration rate (GFR), urinary albumin excretion, and cardiac echo were measured. The animals were then anesthetized to collect blood, and harvest the heart and kidney tissue. The animal protocol of this study has been approved by the IACUC of the University of Colorado Denver and all experimental methods and procedures were carried out in accordance with the approved guidelines.

### Drugs and chemicals

Deoxycorticosterone acetate (DOCA) pellets were purchased from Innovative Research of America, Sarasota, FL, USA. Serelaxin was provided by Novartis.

### Blood pressure measurements

Systolic blood pressure (SBP) was determined by the tail-cuff method using BP-2000 blood pressure analysis system from Visitech Systems (Apex, NC). At least 25–30 measurements per time point were pooled to obtain a mean for each animal.

### Echocardiographic studies

Echocardiographic analyses were performed at the end of the 8-week treatment period using a Vevo770 System equipped with a 30 MHz frequency mechanical transducer (Visual Sonics). Hearts were imaged in the two-dimensional parasternal short axis. M-mode images were recorded to measure LV wall dimensions and internal diameter at the level of the papillary muscles. For analyses, animals were anesthetized using 2% isoflurane and their body temperature was maintained at 37 °C. GraphPad Prism software was used to generate graphs and analyze data. One-way ANOVA with Newman-Keuls post hoc test (P < 0.05) was used to determine statistical differences between groups.

### Urine chemistry

Urine albumin and creatinine concentrations were determined using kits from Exocell (Philadelphia, PA). Rat KIM-1 ELISA kit was purchased from ABCAM (Cambridge, MA).

### Glomerular filtration rate (GFR)

GFR was measured using kits from BioPAL (Worcester, MA).

### RNA extraction and quantitative real-time PCR

Total RNA was isolated from the kidneys using the SV total RNA isolation system from Promega (Madison, WI), and cDNA was synthesized using reverse transcript reagents from Bio-Rad Laboratories (Hercules, CA). The mRNA level was quantified using a Bio-Rad iCyCler real-time PCR machine. 36b4 was used as an internal control, and the amount of RNA was calculated by the comparative threshold cycle (CT) method as recommended by the manufacturer. All the data were calculated from triplicate reactions. Primer sequences are listed in Supplementary Table S1.

### Western blotting

Cortical homogenate protein content was measured by BCA assay (Thermo Fisher Scientific, Waltham, MA). Equal amounts of total protein were separated by SDS-PAGE gels and transferred onto PVDF membranes. The primary antibodies used include NFκB (3033; Cell Signaling Technology, Danvers, Massachusetts; 1:1000 dilution), phospho-NFκB (4764; Cell Signaling Technology, Danvers, Massachusetts; 1:1000 dilution), and β-actin (A5316; Sigma-Aldrich; 1:5000 dilution). After HRP-conjugated secondary antibodies, the immune complexes were detected by chemiluminescence captured on UVP Biospectrum 500 Imaging System (Upland, CA) and the densitometry was performed with ImageJ software.

### Histological stains to assess cardiac and renal fibrosis

After formalin fixation, tissues were processed and embedded into paraffin. 5 μm thick sections were cut onto glass slides. For quantification of the fibrosis following Picrosirius Red (PSR) staining, six 100X polarized light images were made in a “tiling” fashion across each PSR stained cardiac and kidney sections, and then quantified using the 3I Slidebook program (3I, Denver, Colorado) to arrive at the PSR stained pixels per 100x field for that slide.

### Immunofluorescence Microscopy

Kidney sections were stained for the presence of CD68 (monocytes/macrophage marker) (ab955; Abcam, Cambridge, MA; 1:300). Immunoreactivity was visualized using secondary antibodies conjugated with Alexafluor 488 at dilutions of 1:500. Immunofluorescence images were captured on a Zeiss LSM 780 confocal microscope.

### Renal Morphology Quantification

All quantifications were performed in a masked manner. Using coronal sections of the kidney, 30 consecutive glomeruli per rat, six rats per group were examined for evaluation of glomerular mesangial expansion. The index of the mesangial expansion was defined as ratio of mesangial area/glomerular tuft area. The mesangial area was determined by assessment of the PAS-positive and nucleus free area in the mesangium using ScanScope image analyzer (Aperio Technologies, Vista, CA).

### Two Photon Excitation (TPE) and Second Harmonic Generation (SHG) Microscopy

SHG and TPE microscopy for label-free imaging of collagen and related structures was performed using a Zeiss 780 microscope (Carl Zeiss, Jena, Germany) equipped with a Coherent Chameleon Ultra II laser (Coherent, Santa Clara, CA). For SHG, the laser was tuned to 800 nm and laser power of 7% was used throughout all measurements. A filter cube containing a narrow band 390–410 nm emission filter (hq400/20 m-2p, Chroma Technology, Bellows Falls, VT) was used to detect the SHG signal on a non-descanned detector (NDD). A dichroic mirror at 425 nm allows detection of the TPAF signal at 450–700 nm (hq575/250 m-2p, Chroma Technology, Bellows Falls, VT), also with a NDD detector.

### Statistical analysis

Results are presented as means ± SEM. Data were analyzed by one-way ANOVA with post hoc Bonferroni-Dunn (unless other was indicated) for multiple comparisons. Comparisons between two groups were made by unpaired t-test. Statistical significance was accepted at the p < 0.05 level.

## Electronic supplementary material

Supplementary Information
