# Supplementary material for: Serelaxin improves cardiac and renal function in DOCA-salt hypertensive rats
Source: Sci Rep. 2017 Aug 29;7:9793. doi: 10.1038/s41598-017-09470-0 (PMC5574886; doi:10.1038/s41598-017-09470-0)
Supplement: Supplementary file 1 — Supplementary Information [file 41598_2017_9470_MOESM1_ESM.pdf]

Supplemental information:

TITLE: Serelaxin improves cardiac and renal function in DOCA-salt hypertensive rats

Dong Wang[1], Yuhuan Luo[1], Komuraiah Myakala[1], David J. Orlicky[2], Evgenia Dobrinskikh[1], Xiaoxin Wang[1], Moshe Levi[1]

AUTHORS: Departments of Medicine[1]and Pathology[2], Division of Renal Diseases and Hypertension, University of Colorado AMC, Aurora, Colorado 80045

Supplementary Table S1: Primers used for real-time PCR

Gene	Primer sequence
FATP1	F-GGGTTTGCAAGCCAGAGA
	R-CAAAGCAGCCCCAATGAG
ChREBP	F-CGACACTCACCCGCCTCTTC
	R-TTGTTCAGCCGAATCTTGTC
ABCA1	F-ACGAGATTGATGACCGCCTC
	R-AGCATCCACCCCACTCTCTTC
ACOX1	F-CTCACTCGAAGCCAGCGTTA
	R-TTGAGGCCAACAGGTTCCAC
Srebp1	F-TGGCGGGCACTACTTAGGAA
	R-GCAACACTGGCAGAGATCTACGT
36b4	F-AAGGAAGAGTCGGAGGAATC
	R-GGCTGACTTGGTGTGAGG
Srebp2	F-TGACTGGATGATGCCGACTC
	R-GTGACCGAGGAGCGTGAG
NFκB	F-TGCAGAAAGAAGACATTGAGGTG
	R-AGGCTAGGGTCAGCGTATGG
MCP1	F-GGCCTGTTGTTCACAGTTGCT
	R-TCTCACTTGGTTCTGGTCCAGT
ICAM-1	F-GCCCGGAGGATCACAAACGAC
	R-CCTGGGGCTGGCATGTAAGAGT
VCAM-1	F-ACAAAACGCTCGCTCAGATT
	R-GTCCATGGTCAGAACGGACT
Col1A	F-ATCAGCCCAAACCCCAAGGAGA
	R-CGCAGGAAGGTCAGCTGGATAG
Col3A	F-TGATGGGATCCAATGAGGGAGA
	R-GAGTCTCATGGCCTTGCGTGTTT
HMGCR	F-GGTTCTTGTTCACGCTCA
	R-ATTCTCTTGGACACATCTTCA
IL1	F-AATCTCACAGCAGCATCTC
	R-AGCAGGTCGTCATCC
IL6	F-CTTCCAGCCAGTTGCCTTCTTG
	R-TGGTCTGTTGTGGGTGGTATCC
TGFβ1	F-ACCGCAACAACGCAATCTAT
	R-ACGCCAGGAATTGTTGCTAT
LDLR	F-CCAGTGCGGCGTAGGATT
	R-GGGACTCATCGGAGCCAT
α-MHC	F-TGTGGTGCCTCGTTCCA
	R-TTTCGGAGGTACTGGGCTG
β-MHC	F-TTGGCACGGACTGCGTCATC
	R-GAGCCTCCAGAGTTTGCTGAAGGA

ANF	F-ATCTGATGGATTTCAAGAACC
	R-CTCTGAGACGGGTTGACTTC
BNP	F-ACAATCCACGATGCAGAAGCT
	R-GGGCCTTGGTCCTTTGAGA
SERCA	F-GCCTCTTAACCCTGCTGTTG
	R-GGGACTTTTGCTACCAGGTG

Supplemental figure 1. The whole cropped blots presented in figures 4h. Cropped gels blots are used here. PVDF membranes for Western Blot were cut into strips to minimize the amount of antibodies and incubate several antibodies that are necessary for analysis.

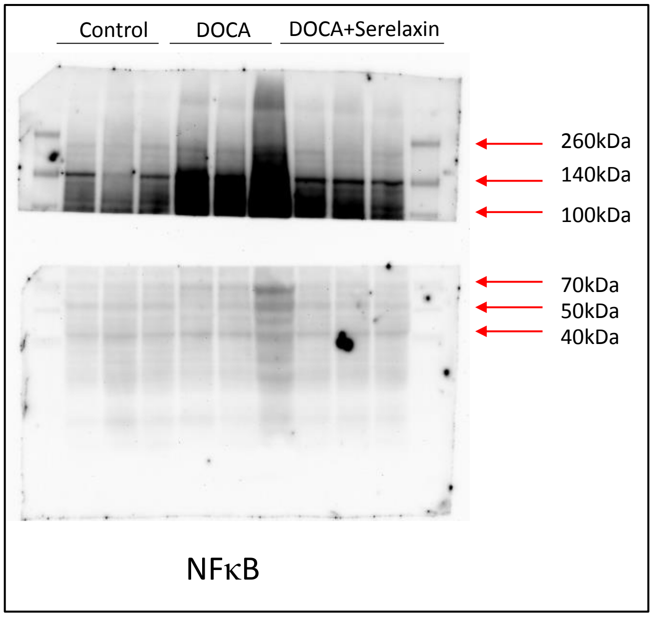

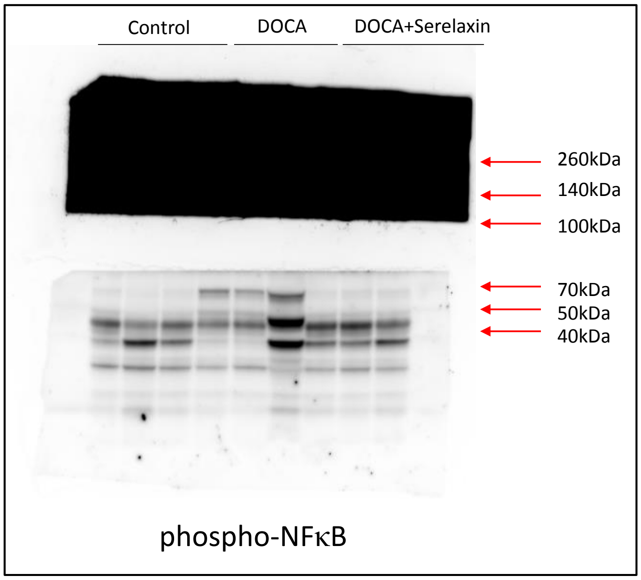
Control
DOCA
DOCA+Serelaxin
260kDa
140kDa
100kDa
70kDa
50kDa
40kDa
phospho-NFκB

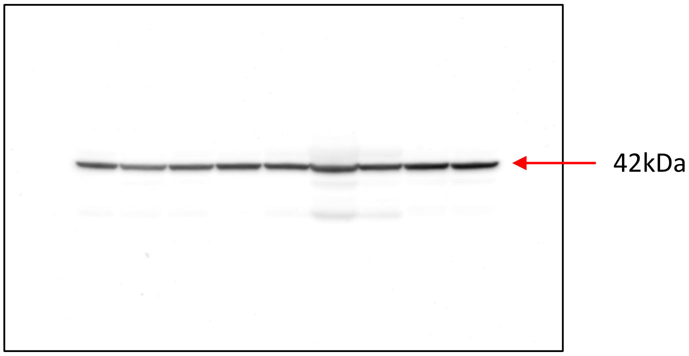
42kDa

β-actin
